# Creation of a *Pseudotsuga menziesii* nucellus cell suspension culture system to produce proteins involved in reproduction

**DOI:** 10.1002/aps3.70048

**Published:** 2026-03-28

**Authors:** Andrea Coulter, YuQi Li, Julia Lakhani, Natalie Prior, Patrick von Aderkas

**Affiliations:** ^1^ Centre for Forest Biology, Department of Biology University of Victoria P.O. Box 3020 Station CSC Victoria V8W 3N5 British Columbia Canada

**Keywords:** gymnosperms, nucellus, plant cell culture, pollination drop, post‐pollination prefertilization drop, proteomics, *Pseudotsuga*

## Abstract

**Premise:**

Cell suspension cultures of Douglas‐fir (*Pseudotsuga menziesii*) nucellus were created to overcome phenological limitations to the in situ study of bioactive proteins within and secreted by nucellar cells.

**Methods:**

Proteins isolated from the cell suspension culture medium were analyzed using mass spectrometry, as were Douglas‐fir post‐pollination prefertilization drops and nucellus tissue excised from the ovule at the time of drop production.

**Results:**

A total of 280 proteins ranging in size from 5 to 224 kDa were found in the three sample types. Identical proteins were found in the post‐pollination prefertilization drop and in the nucellar suspension culture medium or the nucellus. Secretory proteins isolated from the culture medium were either carbohydrate metabolism‐related or defense proteins, consistent with previous proteomic studies of pollination drops. Intracellular proteins were found in all three sample types and were consistent with previous proteomic studies of pollination drops, implying similar metabolic processes are at work in the gymnosperm nucellus at the time of drop production.

**Discussion:**

Cell suspension cultures of Douglas‐fir nucellus are able to produce secretory proteins that are biologically relevant during pollination. This is potentially a tractable system that can overcome barriers to in situ studies presented by gymnosperm reproductive phenology.

Almost all gymnosperm ovules produce a liquid secretion at the time of receptivity. Two forms of the secretion have been observed: the pollination drop, which is secreted simultaneous to pollination, and the post‐pollination prefertilization drop, which is secreted after pollination but before fertilization. The former is more common than the latter, which is restricted to two genera in the Pinaceae, *Pseudotsuga* Carrière and *Larix* Mill. (Gelbart and von Aderkas, 2002; von Aderkas et al., 2018). Both forms of secretion are involved in transporting pollen to the interior of the ovule, making them part of gymnosperm pollination mechanisms. Pollination drops fill and then exude from the micropyle, forming an exposed drop at the micropylar opening. Pollen captured by the drop is brought to the nucellus as the drop retracts. Post‐pollination prefertilization drops fill the micropyle and carry pollen previously captured by collapsing stigmatic hairs to the nucellus as they retract (Coulter et al., 2012; von Aderkas et al., 2018).

Because the drop facilitates pollination, research has generally focused on describing drop morphology and phenology (e.g., Tison, 1911; Doyle, 1945; Singh, 1978; Tomlinson, 1994; Owens et al., 1998; Che et al., 2021). However, a growing body of research is examining the composition of the drop. Calcium (Fujii, 1903; von Aderkas et al., 2012), phosphates (Ziegler, 1959), sugars, and amino acids (Nepi et al., 2017) have been identified in the drop of a number of gymnosperm taxa. Proteins have been identified in the drop of all species studied to date, including multiple conifer species (Poulis et al., 2005; O'Leary et al., 2007; Wagner et al., 2007; Pirone‐Davies et al., 2016), *Welwitschia mirabilis* Hook.f. (Wagner et al., 2007), multiple *Ephedra* L. (von Aderkas et al., 2015) and *Gnetum* L. (Hou et al., 2019) species, and *Cycas revoluta* Thunb. (von Aderkas et al., 2022). This biochemical complexity suggests functions for the drop beyond pollen capture and transport. Exogenous calcium, for example, is essential for pollen germination and pollen tube growth (Brewbaker and Kwack, 1963) as are sugars and amino acids (Nygaard, 1977; Shivanna, 2003), suggesting an important role for the drop in pollen germination. The composition and concentration of sugars is important for successful pollen tube growth and is species‐specific (Shivanna, 2003; Rottman et al., 2018). There is evidence of pollen selection occurring in the drop due to differences in carbohydrate composition (von Aderkas et al., 2012). Sugars and amino acids also serve as pollinator rewards, and pollinator‐driven selection has led to a marked evolutionary difference in sugar and amino acid profiles between the drops of anemophilous and ambophilous gymnosperms (Nepi et al., 2017). Proteins identified to date in the drop are mainly involved in carbohydrate modification (e.g., invertase, beta‐galactosidase) or defense (e.g., chitinase, thaumatin‐like protein) (Poulis et al., 2005; O'Leary et al., 2007; Wagner et al., 2007; von Aderkas et al., 2015, 2022; Pirone‐Davies et al., 2016). Experimental work has confirmed both types of enzymatic activity in the *Pseudotsuga menziesii* (Mirbel) Franco drop: invertase activity is thought to support homospecific pollen germination by regulating drop sugar composition (von Aderkas et al., 2012), and chitinase activity likely prevents fungal contamination by cleaving chitin, a common component of fungal cell walls (Coulter et al., 2012).

The ability to investigate pollination and post‐pollination prefertilization drops is limited by sample availability. The drops are produced once annually for approximately two weeks and are very small, averaging just 60 nL and ranging from 10 nL in *Chamaecyparis lawsoniana* (A. Murray bis) Parl. to 250 nL in *Taxus* L. species (Seridi‐Benkaddour and Chesnoy, 1988; Nepi et al., 2012). The source of the drop is the nucellus, the sporogenous tissue within the ovule that gives rise to the megagametophyte (Nepi et al., 2009). Here, we document the creation of a cell suspension culture system to produce proteins secreted from the nucellus that are biologically relevant during pollination by culturing *P. menziesii* nucellus tissue at the time of post‐pollination prefertilization drop production (Figure 1). We also document the proteins present in *P. menziesii* post‐pollination prefertilization drops and nucellar tissue at the time of drop production.

**Figure 1:**
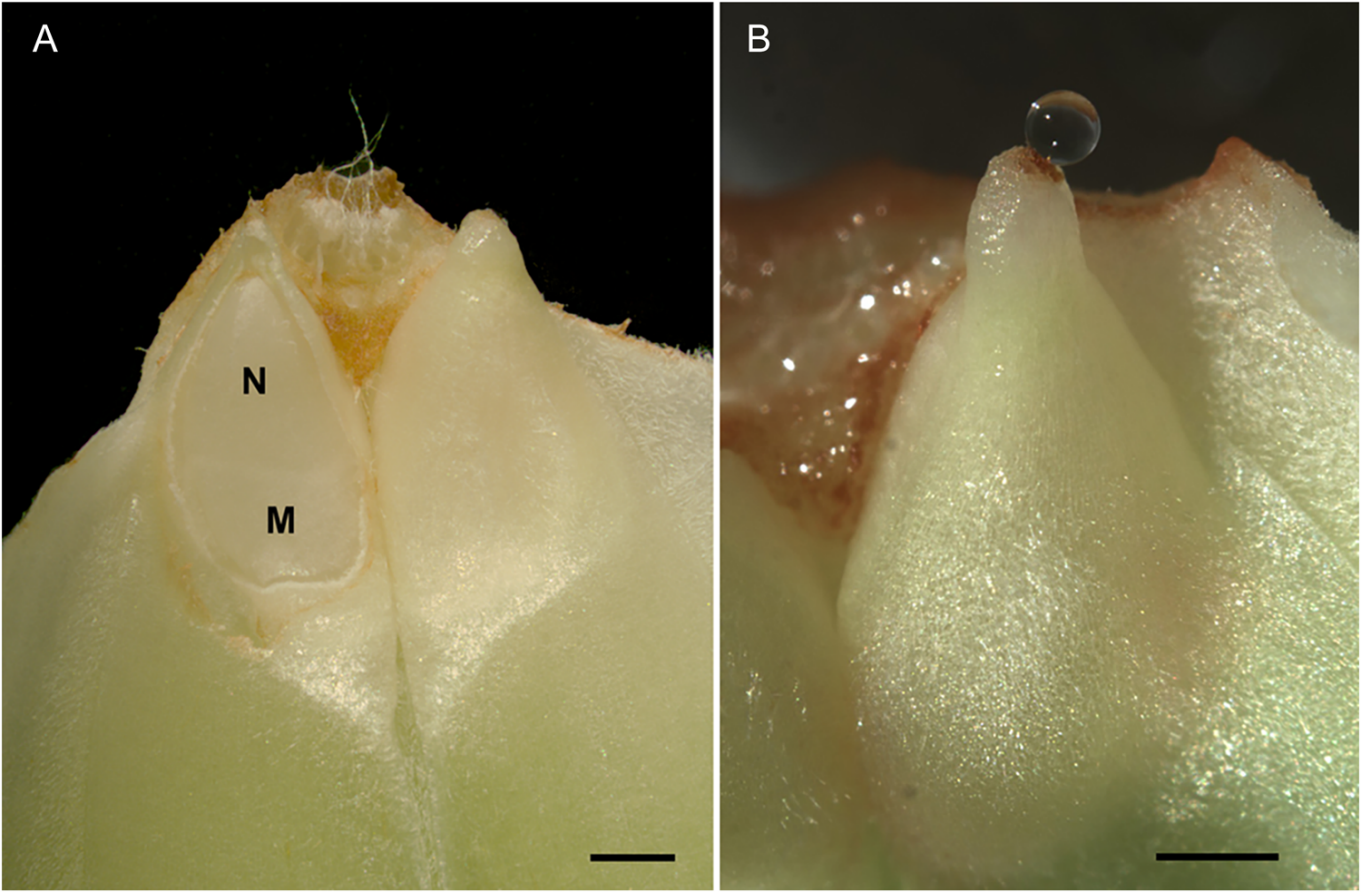
*Pseudotsuga menziesii* ovuliferous scale–bract complexes. (A) The integument has been removed from an ovule to show the nucellus (N) and the megagametophyte (M). (B) A post‐pollination prefertilization drop exuding from the micropylar opening of an ovule. Drops can only be accessed by removal of the ovuliferous scale–bract complexes. Scale bars = 1 mm.

## METHODS

### Nucellus suspension culture

*Pseudotsuga menziesii* (Douglas‐fir) cones were harvested from trees growing at the Mount Newton Seed Orchard (Mosaic Forest Management, Saanichton, British Columbia, Canada). Cone collection was timed to coincide with post‐pollination prefertilization drop production (late May to early June, 6–10 weeks after pollination). To prevent pollination, cones were covered with pollen exclusion bags. Cones were collected from three genotypes per year for two years (year 1: 169, 299, 593; year 2: 38, 299, 327), for a total of five genotypes. The nucellus was excised on the same day as cone collection; cones were stored at 4°C ± 1°C until excision.

Ovuliferous scale–bract complexes were removed from cones, surface sterilized by submerging in 70% ethanol for 5 min and then in a 20% Javex solution (Javex‐12, 12.6% w/v sodium hypochlorite; Colgate‐Palmolive, Toronto, Ontario, Canada) for 15 min, and then rinsed three times in sterile distilled water. The nucellus was then excised from the ovule under sterile conditions, placed on induction medium (five explants per 100 × 15 mm Petri dish), and kept in darkness at 22°C ± 1°C.

A modified Murashige and Skoog (1962) medium was used for induction as outlined in Table 1. Modifications to the inorganic constituents were informed by Becwar et al. (1990). The media was supplemented with sucrose (10 g/L) and solidified with Phytagel (3.2 g/L) (Sigma, St. Louis, Missouri, USA). The pH was adjusted to 5.8 prior to autoclaving for 20 min at 121°C. Glutamine (10 mM), 2,4‐dichlorophenoxyacetic acid (20 µM), and benzylaminopurine (10 µM) were filter sterilized and added to the media following autoclaving.

**Table 1:** Modified Murashige and Skoog media composition.

Component	mg/L
KNO_3_	100
CaCl_2_•2H_2_O	440
MgSO_4_•7H_2_O	370
KH_2_PO_4_	170
KCl	740
KI	0.83
Fe 330 sequestrene	40
H_3_BO_3_	6.2
MnSO_4_•4H_2_O	22.3
ZnSO_4_•7H_2_O	8.6
Na_2_MoO_4_•2H_2_O	0.25
CuSO_4_•5H_2_O	0.025
CoCl_2_•6H_2_O	0.025
Myo‐inositol	100
Nicotinic acid	0.5
Pyridoxine HCl	0.5
Thiamine HCl	0.1

Successfully induced tissue was transferred to a maintenance medium approximately two months after placement on the induction medium. A modified Murashige and Skoog medium was used for the maintenance medium and prepared as for the induction medium, with the exception that plant growth regulators were supplied at half strength (10 µM 2,4‐dichlorophenoxyacetic acid, 5 µM benzylaminopurine). Cultures were kept in darkness at 22°C ± 1°C and subcultured onto fresh maintenance medium every two weeks.

Healthy, friable tissue was transferred to a liquid maintenance medium prepared as for the solid maintenance medium, but with the omission of Phytagel. Approximately 0.7 g of tissue was placed in a sterile 125 mL Erlenmeyer flask containing approximately 25 mL of media. Flasks were plugged with sterilized, cheesecloth‐wrapped cotton plugs and capped with sterilized tin foil. Cultures were kept in darkness at 20°C ± 1°C on an orbital shaker set at 76 rpm. Cultures were first subcultured after 14 days and then subcultured every 10 days. To subculture, approximately 10 mL of media containing suspended cells was combined with approximately 40 mL of fresh media within a sterile 125 mL Erlenmeyer flask and plugged and capped as described above.

### In vitro nucellus proteomics

Proteins were isolated from the liquid culture medium 10 days after cultures were subcultured. Liquid cultures were vacuum filtered (max 10 kPa) through Whatman #1 filter paper (Cytiva, Marlborough, Massachusetts, USA) to remove cellular material. The filtrate was then passed through an Amicon Ultra centrifugal filter with a 3 kDa molecular weight cutoff (MilliporeSigma, Billerica, Massachusetts, USA) at 1310 × *g* and 22°C until the sample had been concentrated by a factor of approximately 100. A single centrifugal filter was used for multiple sample aliquots to minimize protein loss from adherence to the filter membrane. The concentrated sample was stored at −20°C until analysis.

Proteins from three genotypes (38, 299, 327) were run on a one‐dimensional (1D) sodium dodecyl sulfate–polyacrylamide gel electrophoresis (SDS‐PAGE) gel to compare protein profiles. Proteins from a single genotype (299) were processed for mass spectrometry as previously described (Prior et al., 2013; Pirone‐Davies et al., 2016).

Peptide mixtures were rehydrated to 100 µL with 2% acetonitrile/water/2% formic acid and separated by on‐line reversed‐phase chromatography using an EASY‐nLC II system (Thermo Fisher Scientific, Waltham, Massachusetts, USA) with a reversed‐phase pre‐column Magic C‐18AQ and a reversed‐phase nano‐analytical column Magic C‐18AQ (Michrom BioResources, Auburn, California, USA), both prepared in‐house. The chromatography system was coupled to an LTQ Orbitrap Velos mass spectrometer equipped with a Nanospray II source (Thermo Fisher Scientific).

Raw liquid chromatography–mass spectrometry (LCMS) files were converted to Mascot generic format (.mgf) and processed with PEAKS Client 6 (Bioinformatics Solutions, Waterloo, Ontario, Canada) with Peaks DB and SPIDER searches enabled against the UniProt/TrEMBL and 11 UniProt/Swiss‐Prot taxonomy databases as done previously for *Ephedra* (von Aderkas et al., 2015). Proteins were identified that were above a 95% protein and a 95% peptide threshold with a minimum of two unique peptides per protein; the selected proteins had a minimum protein identification probability of 99%. The decoy database percolator settings were max delta Cn 0.05, target FDR strict 0.01, and target FDR relaxed 0.05, with validation based on *q*‐value.

### Post‐pollination prefertilization drop proteomics

Douglas‐fir cones were harvested at the time of post‐pollination prefertilization drop production from trees growing at the University of Victoria (Victoria, British Columbia, Canada). Post‐pollination prefertilization drops were collected from 10 cones, pooled, and stored as previously described (Poulis et al., 2005).

A pooled drop sample (5 μL) was processed and run on a 1D SDS‐PAGE gel and then processed for mass spectrometry as previously described (Prior et al., 2013; Pirone‐Davies et al., 2016). Peptides were separated using a reversed‐phase pre‐column Magic C18‐AQ and an in‐house‐prepared reversed‐phase nano‐analytical column Magic C‐18AQ (Michrom BioResources). The chromatography system was coupled online with an LTQ Orbitrap Velos mass spectrometer equipped with a Nanospray II source (Thermo Fisher Scientific).

Raw files were viewed with Xcalibur 4.0 software (Thermo Fisher Scientific) and analyzed with Proteome Discoverer 1.4.0.228 software (Thermo Fisher Scientific). Peak lists were submitted to an in‐house Mascot 2.4.1 server against UniProt/Swiss‐Prot databases. Statistical analyses of the Proteome Discoverer result files were performed with the Scaffold Q+S software package (Proteome Software, Portland, Oregon, USA). Proteins were identified as described for in vitro nucellus proteomics.

### In vivo nucellus proteomics

Douglas‐fir cones were harvested at the time of post‐pollination prefertilization drop production from trees growing at the University of Victoria (Victoria, British Columbia, Canada) and placed on ice. The nucellus was dissected from the ovule and immediately frozen at −80°C; nucelli were pooled and stored at −80°C until analysis.

Pooled nucellus was ground in liquid nitrogen and then solubilized in 4 M urea in 100 mM ammonium bicarbonate before an overnight cold acetone precipitation. The sample was then spun down and re‐solubilized in the urea/ammonium bicarbonate solution. Proteins were then run on a 1D SDS‐PAGE gel and processed for liquid chromatography–tandem mass spectrometry (LC‐MS/MS) as previously described (Prior et al., 2013; Pirone‐Davies et al., 2016).

Peptide mixtures were separated by a reversed‐phase pre‐column ReproSil‐Pur C18‐AQC18 A1 EASY (Thermo Fisher Scientific) and an in‐house‐prepared reversed‐phase nano‐analytical column Magic C‐18AQ (Michrom BioResources). The chromatography system was coupled on‐line with an LTQ Orbitrap Fusion Tribrid mass spectrometer equipped with a Nanospray Flex NG source (Thermo Fisher Scientific).

Raw files were analyzed using Proteome Discoverer 1.4.0.228 software (Thermo Fisher Scientific) with a Mascot 1.4.1.14 server (Matrix Science, Boston, Massachusetts, USA) against the UniProt/Swiss‐Prot databases. Statistical analyses of the Proteome Discoverer result files were performed with the Scaffold Q+S software package (Proteome Software). Proteins were identified as described for in vitro nucellus proteomics.

## RESULTS AND DISCUSSION

**Figure 2:**
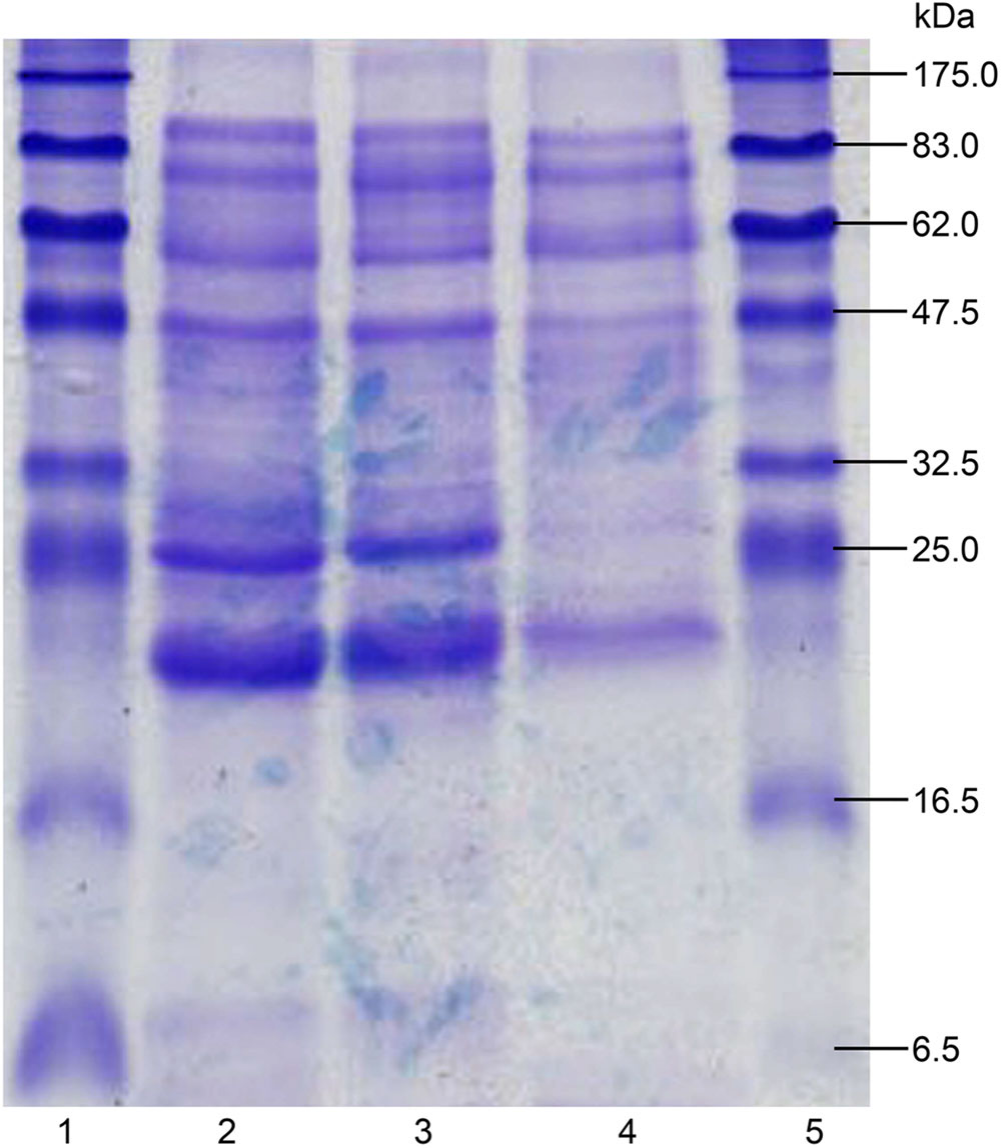
A one‐dimensional SDS‐PAGE gel comparing the proteins isolated from the liquid culture medium of three different genotypes of *Pseudotsuga menziesii* nucellus grown in vitro. Lanes 1, 5: broad‐range molecular weight marker; lane 2: genotype 299; lane 3: genotype 327; lane 4: genotype 38.

Callus tissue was induced to grow from nucellar explants from all five genotypes, with induction rates ranging from 4.44% to 62.9% (Table 2). The tissue was cream colored and friable. Once transferred to solid maintenance medium, cultures were maintained for more than a year without an observable decrease in growth or quality. Callus tissue was also successfully maintained in liquid maintenance medium, in which the callus formed a suspension of free, cream‐colored cells that were evenly distributed throughout the culture medium. Proteins isolated from the liquid culture medium were consistent across genotypes, according to the 1D SDS‐PAGE profiles (Figure 2).

**Table 2:** Induction of callus from *Pseudostsuga menziesii* nucellus explants growing on a modified Murashige and Skoog induction medium.

Year	Genotype	Explants	Induced explants	Induction rate
1	169	50	20	40.0%
	299	50	19	38.0%
	593	45	3	6.67%
2	38	45	2	4.44%
	299	45	6	13.3%
	327	35	22	62.9%

**Figure 3:**
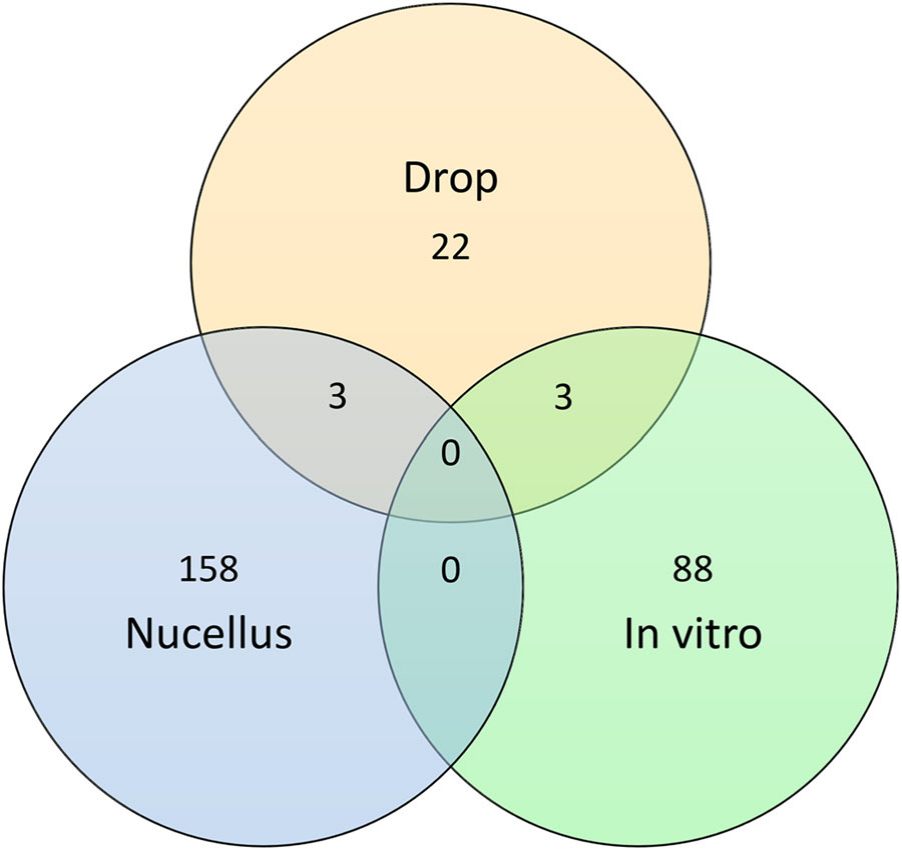
A Venn diagram illustrating the number of proteins identified by mass spectrometry from *Pseudotsuga menziesii* nucellus, the liquid culture medium of *P. menziesii* nucellus grown in vitro, and *P. menziesii* post‐pollination prefertilization drops.

A total of 280 proteins ranging in size from 5 to 224 kDa were identified from the liquid culture medium of Douglas‐fir nucellus grown in vitro, the Douglas‐fir post‐pollination prefertilization drop, and the in vivo Douglas‐fir nucellus (Table 3; for a complete list of identified proteins and their accession numbers, see Appendix S1). Identical proteins were found in the culture medium and the drop and in the nucellus and the drop. No protein was found to be in common between the culture medium and the nucellus or in all three materials sampled (Figure 3, Appendix S1). The nucellus had the greatest protein diversity, the in vitro nucellus culture medium had the second highest diversity, and the post‐pollination prefertilization drops had the least diversity.

**Table 3:** A summary of proteins identified by mass spectrometry from the liquid culture medium of *Pseudotsuga menziesii* nucellus grown in vitro, *P. menziesii* post‐pollination prefertilization drops, and *P. menziesii* nucellus. Secreted proteins are bolded.

Protein	In vitro	Drop	Nucellus
Alpha‐galactosidase	X	X	
Beta‐galactosidase	X	X	
**Beta‐xylosidase/alpha‐l‐arabinofuranosidase 1 (fragment)**	**X**	**X**	
Peptidyl‐prolyl *cis‐trans* isomerase 1		X	X
Thioredoxin H‐type		X	X
Ubiquitin‐NEDD8‐like protein RUB1		X	X
Not in common	88	22	158
Total number of proteins	91	28	161
**Total number of secreted proteins**	**31**	**6**	**0**

Of the 91 proteins identified from the culture medium of Douglas‐fir nucellus grown in vitro, 31 were proteins that contain a signal peptide for secretion into the apoplast (Table 3, Appendix S1). These proteins fall largely into two categories. The first includes carbohydrate metabolism–related proteins such as numerous beta‐galactosidases and beta‐xylosidases, laccases, pectinesterases (e.g., pectinesterase/pectinesterase inhibitor 23) and xyloglucan endotransglucosylase/hydrolase protein A. The second includes defense proteins, such as antifungal protein ginkbilobin‐2, antimicrobial peptide 1, cationic peroxidase, pathogenesis‐related protein PRB1‐2, and defensin‐1. This agrees with past reports of such “secretome” proteins sequenced from drops of Douglas‐fir and other gymnosperm species, where proteins functioning to protect against pathogens and involved in altering cell wall components were found (e.g., Coulter et al., 2012; von Aderkas et al., 2012; Pirone‐Davies et al., 2016; Cheng et al., 2018; Prior et al., 2018).

Many of the proteins identified from the suspension culture medium and the drop were released from cells as they died. These proteins all have cellular functions but are inactive once released into the apoplast and do not contribute metabolically. Such “degradome” proteins are present in almost all pollination drop samples of gymnosperms, as abundant published sequences show for various cycads (Prior et al., 2018; von Aderkas et al., 2022), *Ephedra* spp. (von Aderkas et al., 2015), *Cephalotaxus* Siebold & Zucc. ex Endl. spp. (Pirone‐Davies et al., 2016), and *Ginkgo biloba* L. (Cheng et al., 2018; Prior et al., 2018). Five of the six proteins listed in Table 3 are cytoplasmic proteins. Of the three found in both the nucellus and the post‐pollination prefertilization drop, the first, a thioredoxin, is a class of proteins that are widespread in plants and have many cellular functions including redox regulation, stress responses, defense responses, regulation of embryogenesis, and seed development (Fukushi et al., 2024). They are also known to defend against pathogens (Laloi et al., 2024). The second, peptidyl‐prolyl *cis‐trans* isomerase 1, is involved in protein folding, and the third, ubiquitin‐NEDD8‐like protein RUB1, functions in the recycling of proteins. These two proteins are breakdown products that serve no extracellular role. Two proteins that are found in both the suspension culture medium and the post‐pollination prefertilization drop, alpha‐ and beta‐galactosidase, have roles restricted to cytoplasmic carbohydrate metabolism. These same proteins have been found in the pollination drops of *Cycas revoluta* (von Aderkas et al., 2022).

Apart from the shared proteins that have internal cytoplasmic functions, there were many cellular proteins unique to each sample. The suspension culture medium contained 60 proteins that were cytoplasmic in origin, including actins, glucosidases, heat shock proteins, MADS‐box transcription factors, starch synthases, floral homeotic proteins, and membrane proteins. An additional 158 cellular proteins were identified in the in vivo nucellus tissue. These included proteins involved in transport and movement such as tubulins, actins, and ADP‐ribosylation factor 1; chaperone proteins involved in protein folding; heat shock proteins that mediate stress; and signaling proteins such as Ras‐related proteins (e.g., RABA1F, RIC1). There were also proteins involved in recycling and turnover, such as 26S protease regulatory subunit, as well as proteins regulating activity inside the nucleus, such as histones. In the post‐pollination prefertilization drop protein profile, 22 proteins are not secreted, i.e., have intracellular, not extracellular functions. That so many of these same cytoplasmic proteins have been found in recent proteomic studies of pollination drops of *Cycas revoluta* (von Aderkas et al., 2022), *Cephalotaxus* (Pirone‐Davies et al., 2016), and *Ginkgo biloba* (Cheng et al., 2018) implies that similar metabolic processes are at work more generally in the gymnosperm nucellus at the time of drop production (Cheng et al., 2018) and more specifically during nucellar breakdown in Douglas‐fir (Takaso and Owens, 1996).

The proteins found in the liquid culture medium of Douglas‐fir nucellus grown in vitro were consistent across genotypes. An in vitro suspension culture system that reliably produces biologically relevant molecules has practical applications. Significantly, it overcomes a severe limitation in the experimental study of bioactive molecules within and secreted by nucellar cells. In vivo sampling is extremely restricted by reproductive phenology and compounded by the effect of weather on development. In contrast, sampling from in vitro suspension cultures can be done at any time. This allows easier study of nucellus function, which is proving to be far more complicated than previously thought.

The nucellus is the source of the pollination and the post‐pollination prefertilization drop (Poulis et al., 2005; Nepi et al., 2009). Drop secretion, regulation, and function is complex, involving minerals, signaling molecules, and small organic acids, as well as a variety of proteins (Mao et al., 2022). Broadly, the drop functions in one of two ways: supporting pollen–ovule interactions and defending the ovule from microbial infection. There is evidence, for example, that reactive oxygen species in pollination drops regulate pollen germination (Breygina et al., 2024) while calcium found in pollination drops likely supports pollen germination (von Aderkas et al., 2012). Invertase secreted from the nucellus modifies the carbohydrate composition of the pollination drop, likely supporting pollen selection (von Aderkas et al., 2012). Defense proteins are a conserved component of pollination drops that have withstood microbial adaptations (e.g., Poulis et al., 2005; O'Leary et al., 2007; Coulter et al., 2012; Pirone‐Davies et al., 2016; von Aderkas et al., 2018).

The nucellus generally undergoes localized programmed cell death (PCD) to create a receptive site for pollen, the pollen chamber. This process is poorly understood (Offer et al., 2023). From systems such as barley and rice, we know that MADS‐box proteins, as well as a variety of nucellar proteases, influence nucellar PCD in cereals (Van Hautegem et al., 2015). Transcriptomic study of nucellar PCD in *Ginkgo biloba* suggests that phytohormone signaling is involved, as are calcium and proteases (Li et al., 2019). Phytohormones may also play a role in pollination drop secretion (Che et al., 2021).

The nucellus and pollination/post‐pollination prefertilization drops are integral to gymnosperm reproduction, yet much remains unknown. To date, proteomic analysis of the drops has been directed towards protein discovery; however, functional characterization of the drop constituents is needed to fully understand the role of the drop in prezygotic events and ovule defense. Such work is currently limited by drop volume but becomes more feasible if proteins can be produced in vitro. The nucellus is responsible for drop secretion and is where pollen germinates (von Aderkas et al., 2018). Little is known about the interaction between proteins of the pollen grains and proteins in the drop and the nucellus (Nepi at al., 2009). An in vitro cell suspension culture system that produces biologically relevant proteins secreted by the nucellus can supplement immunohistochemistry studies, transformation protocols, and other cell biological technologies to expand the opportunities to study both the nucellus and the drop and the role of both in pollen–ovule interactions and ovule defense.

This study is the first to show that an in vitro system to study the gymnosperm nucellus is possible. It is a proof‐of‐concept that gymnosperm nucellus cell suspension cultures are able to secrete proteins that are biologically relevant during pollination. This is potentially a tractable system that will remove experimental barriers to in situ studies presented by gymnosperm reproductive phenology. Future studies can work to refine this system to produce proteins involved in gymnosperm reproduction that are of experimental interest.

## AUTHOR CONTRIBUTIONS

A.C. conceived the research. A.C., P.v.A., and N.P. planned and designed the research. A.C., Y.L., and J.L. carried out experimental work. A.C., Y.L., and P.v.A. analyzed the data. A.C. and P.v.A. drafted the manuscript. A.C., N.P., and P.v.A. revised the manuscript. All authors reviewed the manuscript and approved the final version of the manuscript.

## Supporting information


**Appendix S1.** Complete list of proteins identified by mass spectrometry from the liquid culture medium of *Pseudotsuga menziesii* nucellus grown in vitro, *P. menziesii* nucellus, and *P. menziesii* post‐pollination prefertilization drops. Identical proteins were found in two out of three sample types as indicated.

## Data Availability

The proteomic data that supports the findings of this research has been made available as supporting information to this article.

## References

[aps370048-bib-0001] Becwar, M. R. , R. Nagmani , and S. R. Wann . 1990. Initiation of embryogenic cultures and somatic embryo development in loblolly pine (*Pinus taeda*). Canadian Journal of Forest Research 20: 810–817. 10.1139/x90-107

[aps370048-bib-0002] Brewbaker, J. L. , and B. H. Kwack . 1963. The essential role of calcium ion in pollen germination and pollen tube growth. American Journal of Botany 50: 859–865. 10.1002/j.1537-2197.1963.tb06564.x

[aps370048-bib-0003] Breygina, M. , O. Luneva , K. Babushkina , O. Schekaleva , and S. Polevova . 2024. Reactive oxygen species in pollination drops of coniferous plants. Theoretical and Experimental Plant Physiology 36: 761–769. 10.1007/s40626-024-00343-2

[aps370048-bib-0004] Che, W. , D. Mao , T. Zhang , B. Jiang , Z. Lu , and L. Wang . 2021. Phytohormone requirements for pollination drop secretion in *Ginkgo biloba* ovules. Botany 99: 251–260. 10.1139/cjb-2020-0113

[aps370048-bib-0005] Cheng, F. , B. Zhao , B. Jiang , Y. Lu , W. Li , B. Jin , and L. Wang . 2018. Constituent analysis and proteomic evaluation of ovular secretions in *Ginkgo biloba*: Not just a pollination medium. Plant Signalling and Behaviour 13: e1550316. 10.1080/15592324.2018.1550316 PMC6296353 30475662

[aps370048-bib-0006] Coulter, A. , B. A. D. Poulis , and P. von Aderkas . 2012. Pollination drops as dynamic apoplastic secretions. Flora 207: 482–490. 10.1016/j.flora.2012.06.004

[aps370048-bib-0007] Doyle, J. 1945. Developmental lines in pollination mechanisms in the Coniferales. Scientific Proceedings of the Royal Dublin Society 24: 43–62.

[aps370048-bib-0008] Fujii, K. 1903. Über die Bestäubungstropfen der Gymnospermen. Berichte der Deutschen Botischen Gesellschaft 21: 211–217.

[aps370048-bib-0009] Fukushi, Y. , Y. Yokochi , T. Hisabori , and K. Yoshida . 2024. Plastidial thioredoxin‐like proteins are essential for normal embryogenesis and seed development in *Arabidopsis thaliana* . Journal of Plant Research 138: 337–345. 10.1007/s10265-024-01611-7 39708257 PMC11910432

[aps370048-bib-0010] Gelbart, G. , and P. von Aderkas . 2002. Ovular secretions as part of pollination mechanisms in conifers. Annals of Forest Science 59: 345–357. 10.1051/forest:2002011

[aps370048-bib-0011] Hou, C. , R. M. K. Saunders , N. Deng , T. Wan , and Y. Su . 2019. Pollination drop proteome and reproductive organ transcriptome comparison in *Gnetum* reveals entomophilous adaptation. Genes 10: e800. 10.3390/genes10100800 PMC6826882 31614866

[aps370048-bib-0012] Laloi, C. , D. Mestres‐Ortega , Y. Marco , Y. Meyer , and J.‐P. Reichheld . 2024. The *Arabidopsis* cytosolic thioredoxin *h5* gene induction by oxidative stress and its W‐Box‐mediated response to pathogen elicitor. Plant Physiology 134: 1006–1016. 10.1104/pp.103.035782 PMC389923 14976236

[aps370048-bib-0013] Li, D. , D. Wu , S. Li , N. Guo , J. Gao , X. Sun , and Y. Cai . 2019. Transcriptomic profiling identifies differentially expressed genes associated with programmed cell death of nucellar cells in *Ginkgo biloba* L. BMC Plant Biology 19: e91. 10.1186/s12870-019-1671-8 PMC6396491 30819114

[aps370048-bib-0014] Mao, D. , H. Tang , N. Xiao , and L. Wang . 2022. Uncovering the secrets of secretory fluids during the reproductive process in *Ginkgo biloba* . Critical Reviews in Plant Science 41: 161–175. 10.1080/07352689.2022.2066805

[aps370048-bib-0015] Murashige, T. , and F. Skoog . 1962. A revised medium for rapid growth and bio assays with tobacco tissue cultures. Physiologia Plantarum 15: 473–497. 10.1111/j.1399-3054.1962.tb08052.x

[aps370048-bib-0016] Nepi, M. , P. von Aderkas , R. Wagner , S. Mugnaini , A. Coulter , and E. Pacini . 2009. Nectar and pollination drops: How different are they? Annals of Botany 104: 205–219. 10.1093/aob/mcp124 19477895 PMC2710891

[aps370048-bib-0017] Nepi, M. , P. von Aderkas , and E. Pacini . 2012. Sugary exudates in plant pollination. In J. Vivanco and F. Baluška [eds.], Secretions and exudates in biological systems, 155–185. Springer‐Verlag, Heidelberg, Germany.

[aps370048-bib-0018] Nepi, M. , S. A. Little , M. Guarnieri , D. Nocentini , N. Prior , J. Gill , P. B. Tomlinson , et al. 2017. Phylogenetic and functional signals in gymnosperm ovular secretions. Annals of Botany 120: 923–936. 10.1093/aob/mcx103 29045531 PMC5710648

[aps370048-bib-0019] Nygaard, P. 1977. Utilization of exogenous carbohydrates for tube growth and starch synthesis in pine pollen suspension cultures. Physiologia Plantarum 39: 206–210. 10.1111/j.1399-3054.1977.tb04037.x

[aps370048-bib-0020] Offer, E. , S. Moschin , S. Nigris , and B. Baldan . 2023. Reproductive mechanisms in *Ginkgo* and *Cycas*: Sisters but not twins. Critical Reviews in Plant Science 42: 283–299. 10.1080/07352689.2023.2235173

[aps370048-bib-0021] O'Leary, S. J. B. , B. A. D. Poulis , and P. von Aderkas 2007. The identification of two thaumatin‐like proteins (TLPs) in the pollination drop of hybrid yew that may play a role in pathogen defense during pollen collection. Tree Physiology 27: 1649–1659. 10.1093/treephys/27.12.1649 17938097

[aps370048-bib-0022] Owens, J. N. , T. Takaso , and C. J. Runions . 1998. Pollination in conifers. Trends in Plant Science 3: 479–485. 10.1016/S1360-1385(98)01337-5

[aps370048-bib-0023] Pirone‐Davies, C. , N. Prior , P. von Aderkas , D. Smith , D. Hardie , W. E. Friedman , and S. Mathews . 2016. Insights from the pollination drop proteome and the ovule transcriptome of *Cephalotaxus* at the time of pollination drop production. Annals of Botany 117: 973–984. 10.1093/aob/mcw026 27045089 PMC4866313

[aps370048-bib-0024] Poulis, B. D. , S. J. O'Leary , J. D. Haddow , and P. von Aderkas . 2005. Identification of proteins present in the Douglas‐fir ovular secretion: An insight into conifer pollen selection and development. International Journal of Plant Sciences 166: 733–739. 10.1086/431808

[aps370048-bib-0025] Prior, N. , S. A. Little , C. Pirone , J. E. Gill , D. Smith , J. Han , D. Hardie , et al. 2013. Application of proteomics to the study of pollination drops. Applications in Plant Sciences 1: e1300008. 10.3732/apps.1300008 PMC4105296 25202539

[aps370048-bib-0026] Prior, N. , S. A. Little , I. Boyes , P. Griffith , C. Husby , C. Pirone‐Davies , D. W. Stevenson , et al. 2018. Complex reproductive secretions occur in all extant gymnosperm lineages: A proteomic survey of gymnosperm pollination drops. Plant Reproduction 32: 153–166. 10.1007/s00497-018-0348-z 30430247 PMC6500509

[aps370048-bib-0027] Rottman, T. , C. Fritz , N. Sauer , and R. Stadler . 2018. Glucose uptake via STP transporters inhibits in vitro pollen tube growth in a HEXOKINASE1‐dependent manner in *Arabidopsis thaliana* . The Plant Cell 30: 2057–2081. 10.1105/tpc.18.00356 30120167 PMC6181011

[aps370048-bib-0028] Seridi‐Benkaddour, R. , and L. Chesnoy . 1988. Secretion and composition of the pollination drop in the *Cephalotaxus drupacea* (Gymnosperm, Cephalotaxaceae). In M. Cresti , P. Gori , and E. Pacini [eds.], Sexual reproduction in higher plants, 345–350. Springer‐Verlag, Berlin, Germany.

[aps370048-bib-0029] Shivanna, K. R. 2003. Pollen biology and biotechnology. Science Publishers, Enfield, Connecticut, USA.

[aps370048-bib-0030] Singh, H. 1978. Embryology of gymnosperms. Gebrüder Borntraeger, Berlin, Germany.

[aps370048-bib-0031] Takaso, T. , and J. N. Owens . 1996. Post‐pollination prezygotic ovular secretions into the micropylar canal in *Pseudotsuga menziesii* (Pinaceae). Journal of Plant Research 109: 147–160. 10.1007/BF02344540

[aps370048-bib-0032] Tison, P. 1911. Remarques sur les gouttelettes collectrices des ovules des conifers. Mémoires de la Société Linnéenne de Normandie 24: 51–64.

[aps370048-bib-0033] Tomlinson, P. B. 1994. Functional morphology of saccate pollen in conifers with special reference to Podocarpaceae. International Journal of Plant Sciences 155: 699–715. 10.1086/297209

[aps370048-bib-0034] Van Hautegem, T. , A. J. Waters , J. Goodrich , and M. K. Nowack . 2015. Only in dying, life: Programmed cell death during plant development. Trends in Plant Science 20: 102–113. https://doi.org/10/1016/j.tplants.2014.10.003 25457111 10.1016/j.tplants.2014.10.003

[aps370048-bib-0035] von Aderkas, P. , M. Nepi , M. Rise , F. Buffi , M. Guarnieri , A. Coulter , K. Gill , et al. 2012. Post‐pollination prefertilization drops affect germination rates of heterospecific pollen in larch and Douglas‐fir. Plant Reproduction 25: 215–225. 10.1007/s00497-012-0193-4 22806585

[aps370048-bib-0036] von Aderkas, P. , N. Prior , S. Gagnon , S. Little , T. Cross , D. Hardie , C. Borchers , et al. 2015. Degradome and secretome of pollination drops of *Ephedra* . Botanical Review 81: 1–27. 10.1007/s12229-014-9147-x

[aps370048-bib-0037] von Aderkas, P. , N. A. Prior , and S. A. Little . 2018. The evolution of sexual fluids in gymnosperms from pollination drop to nectar. Frontiers in Plant Science 9: e1844. 10.3389/fpls.2018.01844 PMC6305574 30619413

[aps370048-bib-0038] von Aderkas, P. , S. Little , M. Nepi , M. Guarnieri , M. Antony , and T. Takaso . 2022. Composition of sexual fluids in *Cycas revoluta* ovules during pollination and fertilization. Botanical Review 88: 453–484. 10.1007/s12229-021-09271-1 PMC9726676 36506282

[aps370048-bib-0039] Wagner, R. E. , S. Mugnaini , R. Sniezko , D. Hardie , B. Poulis , M. Nepi , E. Pacini , and P. von Aderkas . 2007. Proteomic evaluation of gymnosperm pollination drop proteins indicates highly conserved and complex biological functions. Sexual Plant Reproduction 20: 181–189. 10.1007/s00497-007-0054-8

[aps370048-bib-0040] Ziegler, H. 1959. Über die Zusammensetzung des “Bestäubungstropfens” und den Mechanismus seiner Sekretion. Planta 52: 587–599. 10.1007/BF01914757

